# Prediction of survival prognosis of non-small cell lung cancer by APE1 through regulation of epithelial-mesenchymal transition

**DOI:** 10.18632/oncotarget.8660

**Published:** 2016-04-08

**Authors:** Xi Wei, Qing Li, Ying Li, Wei Duan, Chongbiao Huang, Xiangqian Zheng, Lei Sun, Jingtao Luo, Dong Wang, Sheng Zhang, Xiaojie Xin, Ming Gao

**Affiliations:** ^1^ Department of Diagnostic and Therapeutic Ultrasonography, Tianjin Medical University Cancer Institute and Hospital, National Clinical Research Center of Cancer, Key Laboratory of Cancer Prevention and Therapy, Tianjin, China; ^2^ Cancer Center, Daping Hospital and Research Institute of Surgery, Third Military Medical University, Chongqing, China; ^3^ The Third Department of Breast Cancer, Tianjin Medical University Cancer Institute and Hospital, National Clinical Research Center of Cancer, Key Laboratory of Cancer Prevention and Therapy, Tianjin, China; ^4^ Department of Senior Ward, Tianjin Medical University Cancer Institute and Hospital, National Clinical Research Center of Cancer, Key Laboratory of Cancer Prevention and Therapy, Tianjin, China; ^5^ Department of Biochemistry and Molecular Biology, Tianjin Medical University Cancer Institute and Hospital, Tianjin, China; ^6^ The Department of Otorhinolaryngology and Maxillofacial Oncology, Tianjin Medical University Cancer Institute & Hospital, Key Laboratory of Cancer Prevention and Therapy, Tianjin Cancer Institute, National Clinical Research Center of Cancer, Tianjin, China; ^7^ Department of Thyroid and Cervical Tumor, Tianjin Medical University Cancer Institute and Hospital, National Clinical Research Center of Cancer, Key Laboratory of Cancer Prevention and Therapy, Tianjin, China

**Keywords:** non-small cell lung cancer (NSCLC), APE1, epithelial-mesenchymal transition (EMT), lymph node metastasis

## Abstract

The DNA base excision repair gene APE1 involves in DNA damage repair pathway and overexpression in a variety of human cancers. Analyses of patients with non-small cell lung cancer (NSCLC) suggested that multiple factors associated with prognosis of NSCLC patients. Further investigation showed that APE1 expression was able to predict the progression-free survival and overall survival in patients with NSCLC and correlated with lymph node metastasis. Intriguingly, as a stratification of APE1-141 SNPs in APE1 positive expression, we also found APE1-141 GT/GG was identified as a marker for prediction of poor survival in NSCLC patients. In the *in vitro* experiments, the results showed that when APE1 expression was inhibited by siRNA or AT101 (an APE1 inhibitor), the migration and invasion of NSCLC cells were suppressed. Furthermore, epithelial-mesenchymal transition (EMT) markers was tested to provide evidence that APE1 promoted NSCLC EMT through interaction with SirT1. Using NSCLC xenograft models, we confirmed that AT101 shrank tumor volumes and inhibited lymph node metastasis. In conclusion, APE1 could be a potential target for patients with NSCLC metastasis and AT101 is a potent inhibitor in further treatment of NSCLC patients.

## INTRODUCTION

The non-small cell lung cancer (NSCLC) accounts for approximately 85% of all lung cancer [1, 2]. At present age, NSCLC contributes to the major oncologic public health burden around the world [1]. Patients with NSCLC are prone to lymph node metastasis at the advanced stage [3, 4]. Although systemic lymphadenectomy has been recognized as a vital treatment of NSCLC with lymph node metastasis, millions of death still happens annually [3, 5]. Thus, it is urgent need to clarify the molecular mechanism or to search a potential molecular target in the treatment of NSCLC.

Apurinic/apyrimidinic endonuclease 1 (APE1) have function of DNA damage repair and protein reduction-oxidation [6, 7]. It is also a transcriptional co-activator of numerous pathways, including NF-KB, Myb, HIF-1a, PAX, p53 and AP-1 [7, 8]. Furthermore, APE1 is correlated with the invasion, metastasis and chemotherapy resistance in a variety of cancer [7, 9]. In previous studies, the APE1 polymorphism (APE1-141) is proved to associate with high gastrointestinal toxicity tolerance, but no evidence shows correlation with outcome of patients with advanced NSCLC [10]. Moreover, the APE1-141 GG homozygous genotype protected against lung cancer risk reported by Peng Y et al [10].

In order to acquire motility and invasiveness, epithelial-mesenchymal transition (EMT) involves a shedding by epithelial cells of characteristic morphology and gene expression pattern. The loss of E-cadherin expression associates with EMT to enable carcinoma cells to become invasive [11]. APE1 was also found to regulate transforming growth factor β-dependent manner (TGFβ) to promote EMT in human osteosarcoma [9]. Thus, it raises the hypothesis that the role of APE1 in regulation of EMT progress of NSCLC assumes to be clarified.

In our study, we retrospectively study the association between APE1 expression or SNPs and survival prognosis in NSCLC in a Chinese cohort. We then further explore the role of APE1 in regulation of EMT-mediated metastasis in NSCLC.

## RESULTS

### The characteristics of patients with NSCLC involved in lymph node metastasis

The clinical outcomes of patients with NSCLC were analyzed after surgery based on the final pathological diagnosis. As shown in Table 1, the total of 423 cases was grouped into lymph node involvement positive and negative. We found that patients with lymph node metastasis were associated with high TNM stage (III and IV) (*P*=0.000). The toxicity (grade 3-4) from chemotherapy in the treatment of patients with NSCLC was correlated with lymph node involvement (*P*=0.037). Thus, the TNM stage and toxicity are regarded as two import factors in NSCLC with lymph node metastasis.

**Table 1:** The basic characteristics of patients with NSCLC involved in lymph node metastasis

Variable	Lymph node metastasis		χ ^2^	p value
	positive	negative		
**Number (%)**	305(100%)	118(100%)		
**Age(y)**				
<60	173 (51.5)	68(49.5)	0.028	0.866
≥60	132 (48.5)	50(50.5)		
**Gender**				
Male	229(62.2)	87(59.8)	0.082	0.774
Female	76(37.8)	31(40.2)		
**Smoking**				
No	119 (67.4)	50(64.7)	0.400	0.527
Yes	186 (31.1)	68(23.1)		
**Pathological types**				
Squamous carcinoma	90(29.5)	30(25.4)	3.773	0.152
Adenocarcinoma	164(53.8)	75(63.6)		
Other	51(16.7)	13(11.0)		
**TNM stage**				
II	23(7.5)	42(25.6)	76.983	0.000*
III	164(53.8)	17(14.4)		
IV	118(38.7)	59(50.0)		
**Distant metastasis**				
0	190(62.3)	60(50.8)	5.158	0.076
1	67(22.0)	37(31.4)		
≥2	48(15.7)	21(17.8		
**Chemotherapy regimen**				
TP	237(77.7)	99(83.9)	1.998	0.158
GP	68(22.3)	19(16.1)		
**Treatment response**				
CR+PR	63(20.7)	22(18.6)	0.214	0.643
SD+PD	242(79.3)	96(81.4)		
**Toxicity**				
NO	173(56.7)	80(67.8)	4.342	0.037*
Grade 3–4 toxicity	132(43.3)	38(32.2)		

Abbreviations: TP: paclitaxel+cisplatin; GP: gemcitabine+cisplatin; CR: complete response; PR: partial response; SD: stable disease; PD: progressive disease. *, p<0.05

### The univariate and multivariate analysis of prognostic factors in patients with NSCLC

**Figure 1:**
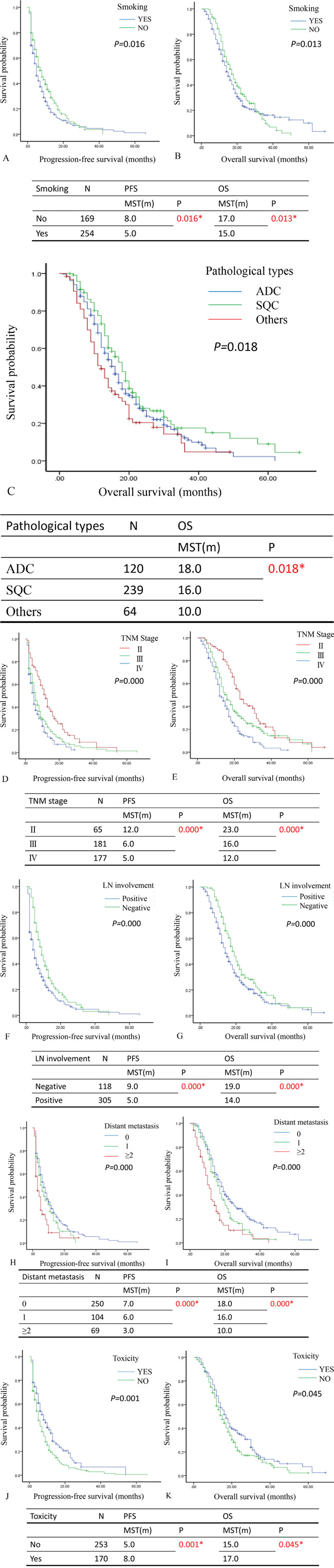
The relationship between survival time and univariate analysis in patients with NSCLC after surgery **A** and **B.** The Kaplan-Meier plot showed the progression-free survival (PFS) and overall survival (OS) in comparison of patients with or without smoking. The patients with smoking associated with poor PFS and OS (*P*=0.016 and *P*= 0.013). **C.** The Kaplan-Meier plot showed the overall survival (OS) in comparison of patients in different type of pathology (ADC, SQC and others). The patients with ADC associated with good PFS and OS (*P*=0.018). **D** and **E.** The Kaplan-Meier plot showed the progression-free survival (PFS) and overall survival (OS) in comparison of patients with different TNM stage (II, III and IV). The patients with TNM stage IV associated with poor PFS and OS (*P*=0.000 and *P*= 0.000). **F** and **G.** The Kaplan-Meier plot showed the progression-free survival (PFS) and overall survival (OS) in comparison of patients with or without lymph node involvement. The patients with lymph node involvement associated with poor PFS and OS (*P*=0.000 and *P*= 0.000). **H** and **I.** The Kaplan-Meier plot showed the progression-free survival (PFS) and overall survival (OS) in comparison of patients with different number of distant metastasis (0,1 and ≥2). The patients with distant metastasis (≥2) associated with poor PFS and OS (*P*=0.000 and *P*= 0.000). **J** and **K.** The Kaplan-Meier plot showed the progression-free survival (PFS) and overall survival (OS) in comparison of patients with or without toxicity. The patients with toxicity associated with poor PFS and OS (*P*=0.001 and *P*= 0.045). ADC: adenocarcinoma; SQC: Squamous carcinoma; LN: lymph node; The median survival time: MST; month: m; number: N; * *P*<0.05.

To further investigate the prognostic factors in NSCLC, univariate and multivariate analyses were performed in progression-free survival (PFS) or overall survival (OS) (Supplemental Table 1). The results of univariate analysis showed that smoking (Figure 1A&1B), pathological types (Figure 1C), TNM stage (Figure 1D&1E), lymph node involvement (Figure 1F&1G), distant metastasis (Figure 1H&1I) and toxicity of chemotherapy (Figure 1J&1K) were independent markers in the prediction of survival time (PFS or OS). Additionally, the multivariate analyses demonstrated the smoking, TNM stage, lymph node metastasis, distant metastasis and toxicity contributed to good factors for PFS (*P*<0.05). The lymph node metastasis, TNM stage III and IV, distant metastasis (1 or ≥2) and toxicity (grade 3-4) are positive prognostic factors of OS to the NSCLC patients (*P* <0.05) (Supplemental Table 2).

### The APE1 expression and polymorphism were detected as makers for patients with NSCLC in lymph node metastasis

**Figure 2:**
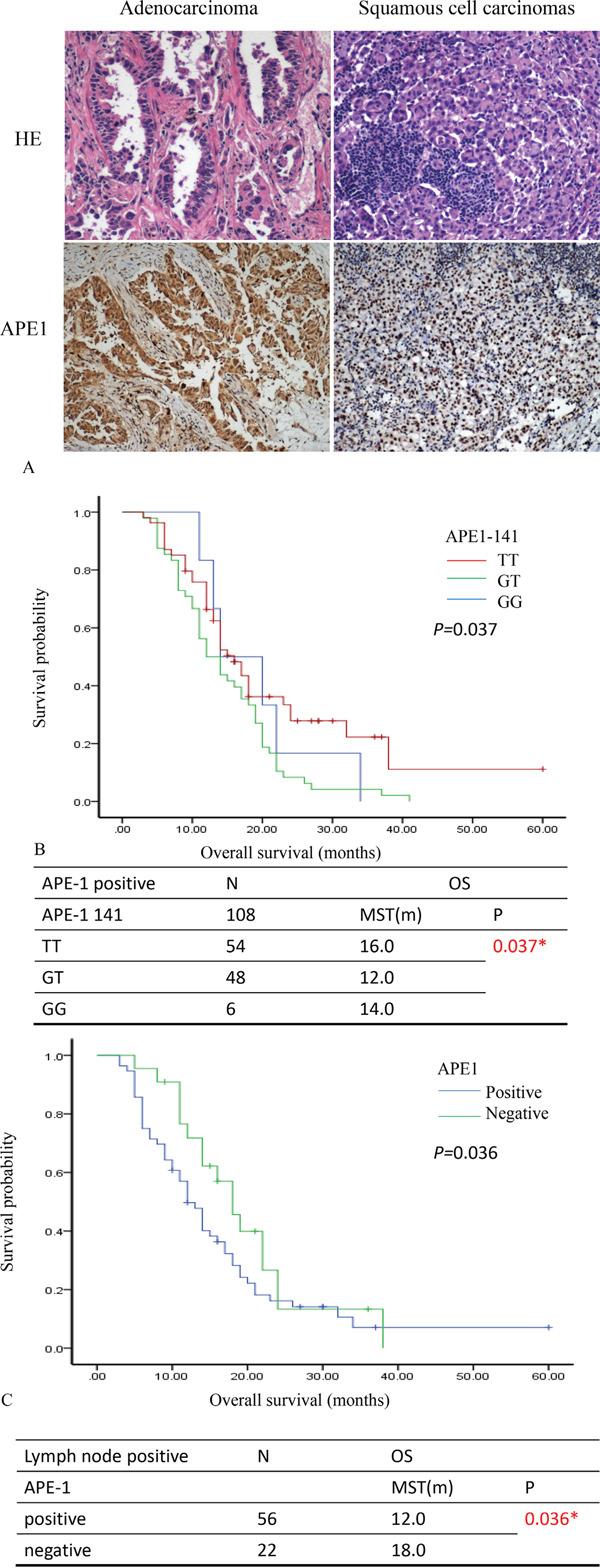
APE1 expression predicts survival prognosis of patients with NSCLC **A.** The samples of patients with NSCLC were stained by Hematoxylin & Eosin (HE) and immunohistochemistry. The expression of APE1 showed in the nucleus and cytoplasm of lung cancer cells. **B.** The comparison of overall survival for polymorphism of APE1-141 stratified by the APE1 expression. The Kaplan-Meier plot showed the overall survival (OS) in comparison of patients with APE1 positive expression in various types of APE1 polymorphism (TT, GT and GG). **C.** The comparison of PFS and OS for the expression of APE1 stratified by the Lymph node metastasis. The Kaplan-Meier plot showed the overall survival (OS) in comparison of patients with lymph node positive in the APE1 expression. The median survival time: MST; month: m; number: N; * *P*<0.05.

First, we stained the APE1 expression in NSCLC patient samples (Figure 2A). The correlation between APE1 expression and lymph node metastasis in NSCLC was indicated by Spearman's rank analysis (r=0.325, *P*=0.000) (Table 2). We also measured the polymorphism of APE1 (APE1 141T/G) to further predict the survival time of patients with NSCLC. The univariate analysis of APE1 141 and APE1 expression separately failed to predict clinical outcome (PFS and OS) (Supplemental Table 3). However, the association of APE1-141 polymorphisms with PFS and OS for patients who were stratified by the APE1 expression was analyzed (Supplemental Table 4). As shown in Figure 2B, the plot showed that patients with positive APE1 whose APE1-141 polymorphisms with GT and GG mutation, have poor prognosis for overall survival (MST: 12.0 months, 14.0 months; *P*=0.037). As for the expression of APE1 stratified by the lymph node metastasis, patients with lymph node metastasis in APE1 positive cases have poor prognosis for OS (MST: 12.0 *vs.*18.0 months; *P*=0.036) (Figure 2C) (Supplemental Table 5). Therefore, combining APE1 expression with SNPs provides clues for further prediction of survival in NSCLC with lymph node metastasis.

**Table 2:** The correlation between APE1 expression and lymph node metastasis in NSCLC patients

Variable	Lymph node involvement			r value	p value
APE1	positive	negative	Total		
Positive	56	31	87	0.325	0.000*
negative	22	14	36		
Total	78	45			

### Inhibition of APE1 attenuated NSCLC invasion and migration *in vitro*

**Figure 3:**
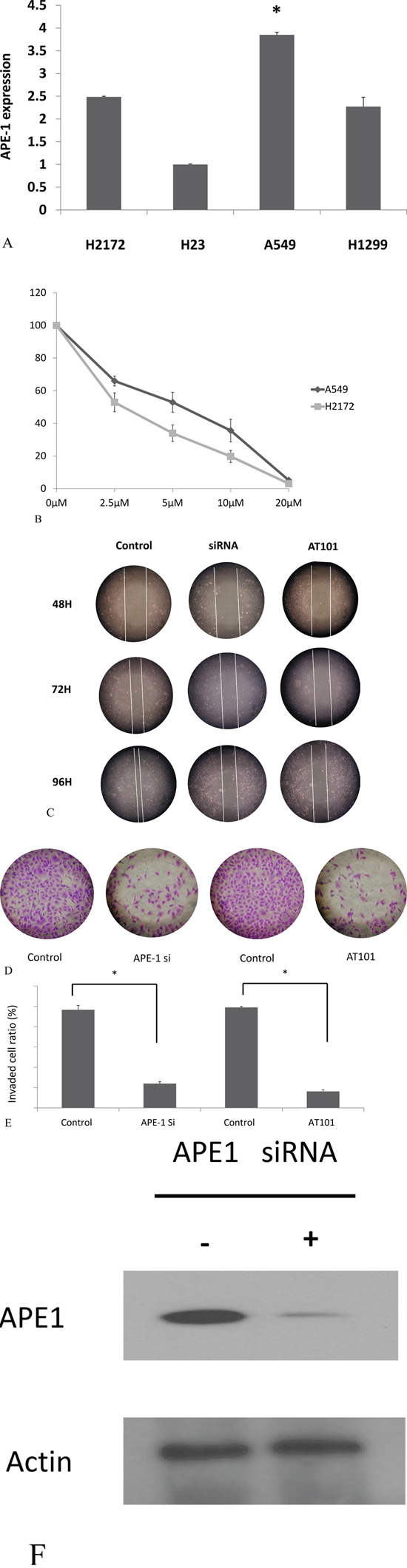
APE1 inhibition promoted non-small lung cancer cells migration and invasion **A.** APE1 expression in H2172, A549, H23 and H1299 cells was measured by RT-PCR; **B.** The five different concentrations of AT101 in the treatment of 48 hours was tested to find an optimal condition in MTT assay **C.** APE1 siRNA or AT101 inhibited A549 cell migration determined by wound healing assay. **D** and **E.** APE1 siRNA or AT101 inhibited A549 cell migration determined by transwell assays with matrigel. * P<0.05. **F.** Knockdown of APE1 by APE1 siRNA as shown.

To test the role of APE1 in the invasion and migration in NSCLC, we first detect the APE1 expression in A549, H2172, H23 and H1299 cells by PCR. A549 cells was showed to be higher expression than other 3 cell lines (*P*<0.05, Figure 3A). Thus, A549 was chosen for further *in vitro* studies. AT101, as a BH3 mimetic and pan-BCL-2 inhibitor, contributes to potent the potential anticancer role in inhibition of APE1/IL-6/STAT3 pathway [15]. APE1 siRNA and AT101 (an APE1 inhibitor) were both used as inhibitors of APE1 expression in A549 cells (Figure 3F). The concentration of 5μM AT101 in the treatment of 48 hours in A549 cells (IC_50_=5μM) was tested as an optimal condition in MTT assay (Figure 3B). As the wound healing assay shown, inhibition of APE1 expression by siRNA or AT101 suppressed the migration ability compared with control siRNA transfected cells or DMSO negative treatment (*P*<0.05, Figure 3C). The transwell assay revealed that APE1 knockdown or inhibition by AT101, A549 cells decreased dramatically in the invasive ability (*P*<0.05, Figure 3D and 3E). Overall, inhibition of APE1 dampens the ability of invasion and migration of NSCLC *in vitro*.

### Inhibition of APE1 contributed to suppression of NSCLC EMT *in vitro*

**Figure 4:**
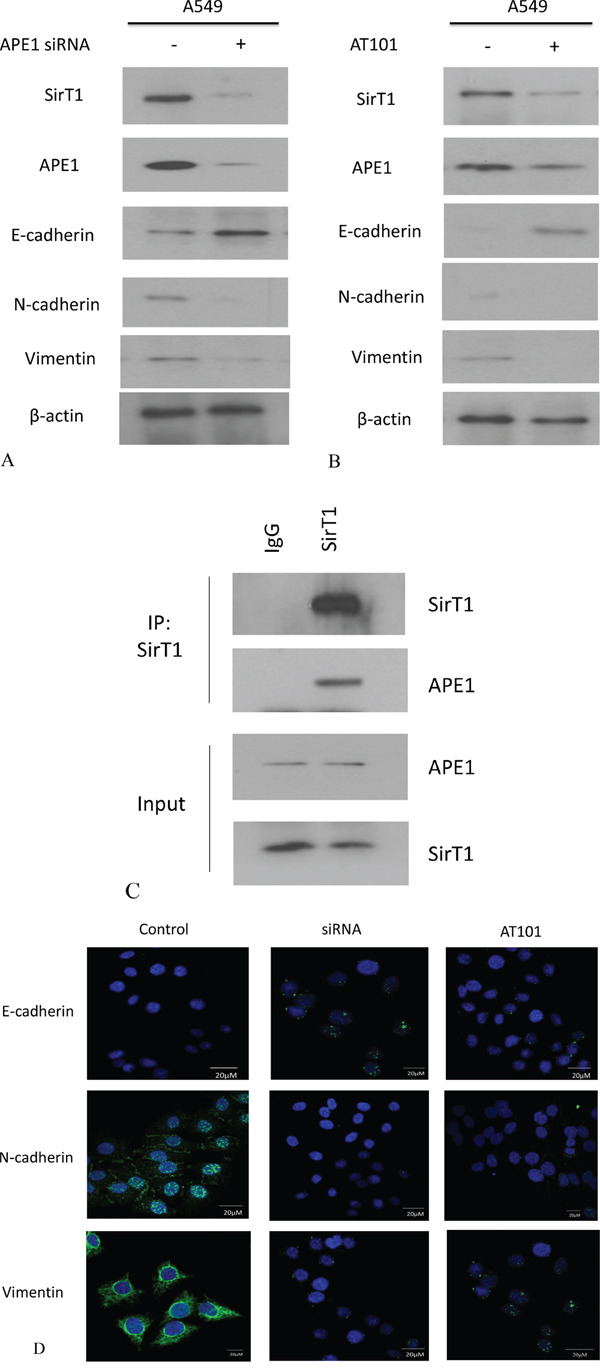
APE1 expression was inhibited by siRNA or AT101 to suppress EMT in NSCLC *in vitro* **A.** APE1 siRNA significantly increased the expression of E-cadherin and inhibited the expression of Vimentin or SirT1 by western blot analysis. **B.** The inhibitor of APE1 – AT101 upregulated the expression of E-cadherin and depressed the expression of Vimentin or SirT1 by western blot analysis. **C.** Endogenous APE1 was pulled down by SirT1 which was immobilized using specific anti-SirT1 antibody in Co-IP assay. **D.** APE1 siRNA and inhibitor AT101 significantly suppressed E-cadherin and downregulated N-cadherin and Vimentin in A549 cells indicated by immunofluorescence. (magnification: 1000×)

The development of EMT in cancer cell relies on the plastic transition between epithelium and mesenchyme. To test whether APE1 involves in NSCLC EMT, we consequently measured the EMT makers (E-cadherin and Vimentin) after inhibition of APE1 expression by siRNA or AT101. We found that the level of E-cadherin increased but Vimentin expression was suppressed after knockdown of APE1 in A549 (Figure 4A and 4B). We also found that inhibition of APE1 was determined by down regulation of sirtuin1 (SirT1) by western blot analysis. Further data suggested that using specific SirT1 antibody enabled to pull down APE1 protein in A549 cells (Figure 4C). The nine potential deacetylated sites (3,6,7,24,25,27,31,32,35) were predicted on N terminal lysines of APE1 in our study, consistence with part of deacetylation sites in previous studies [16, 17] (Supplemental Table 6). Additionally, comparing to negative control, silence of APE1 resulted in increased expression of E-cadherin and decreased expression of Vimentin by immunofluorescence staining (Figure 4D). Taken together, we conclude that inhibition of APE1 suppresses the EMT probably through binding with SirT1 in non-small lung cancer cells.

### APE1 expression presented in lymph node metastasis in NSCLC xenografted mice

**Figure 5:**
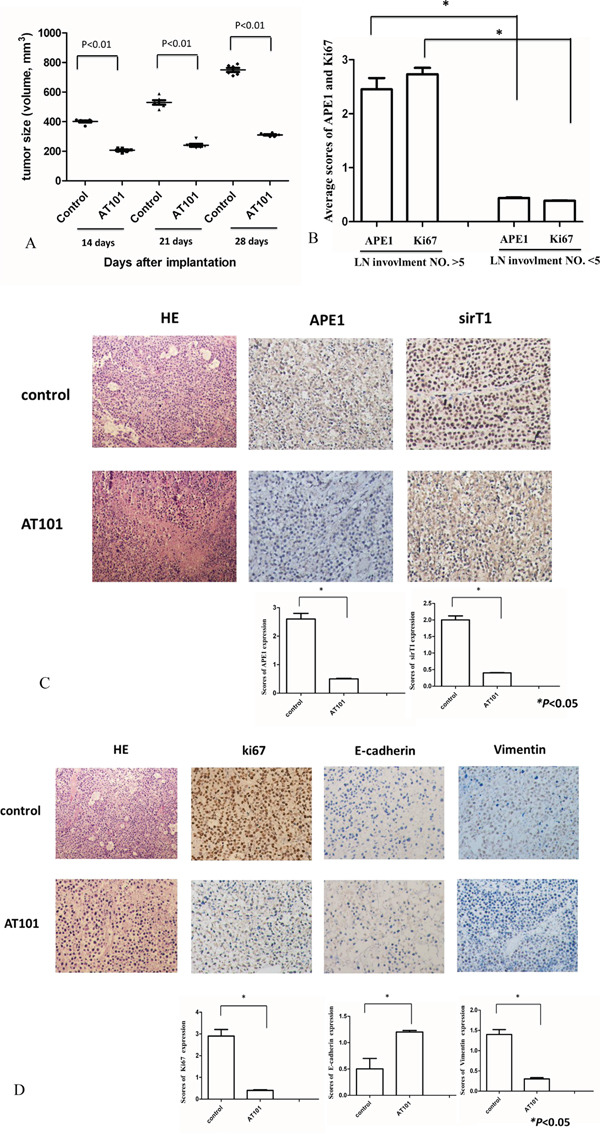
Tumor growth was inhibited by APE1 inhibitor-AT101 *in vivo* **A.** Tumor volumes were shrunk significantly after AT101 treatment (*P*<0.05). **B.** The average scores of APE1 and Ki67 were calculated to be compared within two groups with number of lymph node (LN) involvement >5 or <5 (* *P*<0.05). **C.** After sacrificing, SirT1 expression in xenograft tumors correlated with APE1 status after treatment AT101. Hematoxylin and eosin (HE) staining as control. (magnification: 200×) (* *P*<0.05) **D.** Tumors were stained with EMT and proliferation markers: E-cadherin, Ki-67 and Vimentin by IHC to show their expression in A549 xenograft treated by AT101. Hematoxylin and eosin (HE) staining as control. (magnification: 200×) (* *P*<0.05)

To further explore the role of APE1 in lymph node metastasis and the therapeutic potential of APE1 inhibitor (AT101) in NSCLC, we established xenograft tumor models using A549 cell lines. The volume and weight of tumors described in Figure 5A indicated that, AT101 suppressed tumor growth in mice models, comparing with control or untreated group (*P*<0.05). The further stained APE1 and Ki67 of xenograft tumors suggested the positive correlation between APE1, Ki67 expression and lymph node metastasis in comparison of control and treatment group (Figure 5B). Both of APE1 and SirT1 expression in xenograft tumors were observed as positive correlation after treatment of AT101 (Figure 5C). The other EMT and proliferation markers including E-cadherin, Vimentin and Ki67 were stained by IHC in mice model tumors as well (Figure 5D). Together, APE1 expression promotes NSCLC lymph node metastases and AT101 may serve as an inhibitor in the treatment of NSCLC.

## DISCUSSION

The lymph node involvement is an important factor in influencing the prognosis and therapeutic strategies in NSCLC [15, 18]. The assessment of lymph node status plays a critical role in determining accurate staging and potential survival benefit [18]. In this study, we found that lymph node involvement was correlated with TNM stage or chemotherapeutic toxicity and also considered as an independent factor in prediction of survival of NSCLC patients. APE1′s redox activity acted on regulating the expression of a large number of DNA repair protein and can be activated in DNA damage repair process. The APE1 levels are consistent with the activity of DNA base excision repair under oxidative stress [19, 20]. Moreover, APE1 has been studied as a predictor of cisplatin resistance in NSCLC [10]. The data in our study revealed that APE1 positive expression associated with lymph node metastasis in NSCLC cases. After stratifying APE1-141 polymorphisms in APE1 positive group, the results indicated that patients with GT/GG mutation of APE1 141 had lower overall survival compared to APE1 polymorphisms with T allele. Thus, we predicted that APE1 overexpression and its SNPs link to poor prognosis in NSCLC due to lymph node metastasis.

AT101 is a BH3mimetic and pan-Bcl-2 inhibitor and has presented a potent anticancer activity in NSCLC [15]. To further illuminate the role of APE1 in the regulation of invasive capability of NSCLC, we tested inhibition of APE1 by siRNA or AT101 has ability to suppress lung cancer invasion and metastasis. The small interfering RNA of APE1 was used to enhance the sensitivity of human osteosarcoma cells, according to Wang D's study [9]. Furthermore, silencing APE1 increased E-cadherin and decreased Vimentin expression resulting in EMT suppression. Interestingly, we found that sirtuin-1 (SirT1) expression was reduced after knockdown of APE1 *in vitro*. Antoniali G et al reported that N-terminus of APE1 (1-35 residues) stably binds with SirT1 promoter to regulate its function [21]. The sites on K residues of APE1 (K^6^/K^7^/K^27^/K^31^/K^32^/K^35^) were reported to be deacelyrated by SirT1, consistence with the sites which predicted in our study [17, 22]. It is possible that APE1 is deacetylased by SirT1 by forming a multiprotein complex to response genotoxic stress, leading to APE1 further regulation of EMT. Therefore, APE1 probably promotes the migration of cancer cell to invade in draining lymph nodes through EMT in NSCLC.

In the *in vivo* experiments, using AT101 in the treatment of a NSCLC xenograft model, we revealed that tumor growth and the number of lymph node metastasis were shrunk by AT101. Additionally, the results showed APE1 and Ki67 expression in xenograft tumors were associated with lymph node involvement *in vivo*. The positive correlation between APE1 and SirT1 expression in xenograft tumors supported the results *in vitro*. If it is this case, we speculated that AT101 is a potential inhibitor to NSCLC with lymph node metastasis by inhibiting APE1 expression.

In summary, APE1 and APE1 141 SNPs were implicated as potential markers to predict lymph node metastasis or survival time in patients with NSCLC. For investigation of APE1 function, we demonstrated APE1 promotes lung cancer cells migration and invasion, and subsequently EMT in NSCLC. Furthermore, AT101 can be considered as a potent inhibitor of APE1 expression for further treatment of NSCLC patients with metastasis.

## MATERIALS AND METHODS

### Study population

The total of 423 patients with NSCLC (stage II-IV) was enrolled in this study at Tianjin Medical University Cancer Institute and Hospital (Tianjin, China) and Da ping Hospital, Third Military Medical University (Chongqing, China) between July 2008 and July 2012. The eligible patients were enrolled according to the following criteria: patients did not receive previous chemotherapy or radiotherapy and did not have other malignancy in 5 years before this study; patients with spinal compression, pregnancy, lactation, serious infection or impairment of organ functions were excluded. All patients were diagnosed with squamous cell carcinoma (SQC), adenocarcinoma (ADC) or other histologic types of NSCLC according to WHO classification. 120 (28.4%) with SQC, 239 (56.5%) patients were diagnosed with ADC and 64 (15.1%) with other histology.

Of these 423 patients, 123 were detected immunohistochemistry and polymorphisms of APE1 (Apurinic/apyrimidinic endonuclease. Patients were followed up after surgery by serial clinical examination including tumor markers and thoracoabdominal computed tomography scanning (every 6 months for the first 2 years). The progression-free survival (PFS) and overall survival (OS) were later calculated from the date of surgery until either the time of death or the end of follow-up. This study was approved by the ethics committees of Tianjin Medical University Cancer Institute and Hospital and informed consent was obtained from each participant.

### Genotyping of SNP

APE1 SNPs (141T/G; in the promoter region) were genotyped in all study samples. Genetic polymorphisms were analyzed using PCR-CTPP (PCR with confronting two-pair primers) method as described earlier [12]. Primer pairs and product lengths were designed for each allele and the allele was distinguished based on the SNPs in Base-excision Repair Genes. The primers were added into the same tube. The APE1- 141T/G primers were F1: 5′-CTA ACT GCC AGG GAC GCC GA-3′, R1:5′-ACA CTG ACT TAA GAT TCT AAC TA-3′FOR T allele size of PCR products (136bp); F2:5′- ACT GTT TTT TTC CCT CTT GCA CAG-3′ R2:5′-TGA GCA AAA GAG CAA CCC CG-3′ for G allele size of PCR products (335bp). Which was designed based on the GenBank reference sequence. PCR amplification was performed and PCR products were analyzed via agarose gel electrophoresis. The genotype results were regularly confirmed by randomly selecting 5% of the samples to directly measure DNA sequencing. All genotype frequencies in the control population were in agreement with those predicted under Hardy-Weinberg Equilibrium (*P*>0.05).

### Cell culture and siRNA transfection

Cell lines A549, H23, H2172, H1299 were maintained in Dulbecco modified Eagle medium (DMEM) (Thermo Scientific, USA) with 10% fetal bovine serum (FBS, Thermo Scientific, USA) incubated with 5% CO_2_ at 37°C. Cells were transfected with 100 nM control APE1 siRNA. Sequences of the double-stranded siRNAs are antisense (5′-GUCUGGUACGACUGGAGUACC-3′, 5′-UACUCCAGUCGUACCAGACCU-3′) and nonsense (5′-CCAUGAGGUCAGCAUGGUCUG-3′,5′-GACCAUGCUGACCUCAUGGAA-3′), using RNAiMAX (Invitrogen), according to instructions from the supplier.

### Western blot assay

Cells were lysed in lysis buffer (ZSGB-Bio), centrifuged for 10 minutes at 14,000 x g and the insoluble debris was discarded. Equal amounts of cell lysate (20 μg per lane) were resolved by SDS-PAGE (sodium dodecyl sulfate poly-acrylamide gel electrophoresis) and then transferred to an immobilion P filters (Millipore). The membrane was blocked for 1 h with phosphate-buffered saline (PBS) containing 5% nonfat dry milk and 0.1% Tween 20, incubated with primary and secondary antibodies. The following antibodies were used for western blot assay: APE1 (Cell signaling, MA), 10E4 (Sirtuin1 antibody), β-Actin (all from Santa Cruz, CA), E -cadherin, Vimentin (all from Cell signaling, MA).

### Wound healing assay

A total of 10^4^ A549 cells were seeded in 6-well culture plates after 24 hours growth. When reaching 70~80% confluence as a monolayer, gently and slowly scratch the monolayer using a 200 μL tip across the center of the well. After scratching, 6-well was gently washed twice with PBS to remove the detached cells. The Cells were grown to expose to scramble, APE1siRNA and AT101 for additional 48 h. The fixed cells were stained with 1% crystal violet in 2% ethanol for 30min. The stained monolayer was taken photos for on a microscope which evaluated using Photoshop.

### Transwell assay

Cell migration assay was performed using Corning Matrigel invasion chamber in 24-well plates (Biocoat, MA). Cells were seeded at a density of 30,000 cells per well and then exposed to scramble, APE1siRNA and AT101. The upper chamber was filled with medium without serum, while the lower chamber filled with 20% FBS medium. After 48 h, cells were fixed with 4% formaldehyde and methanol, following with scraping off non-migration cells with cotton swabs. The number of migrated cells was counted under an inverted microscope.

### Immunofluorescence staining

APE1 siRNA transfected A549 and control cells were incubated with primary antibodies (1:200 dilutions) overnight at 4°C and then incubated with Fluorochrome-conjugated secondary antibody (1:100 dilutions, cell signaling, MA) for 1 h at room temperature in the dark. Cell nuclei were coversliped with Gold Anitfade reagent with DAPI. The number of positive cells was counted using LSM780NLO Two-photon microscopes (Carl Zeiss, Germany).

### NSCLC xenograft tumor model

The experimental protocol in nude mice was approved by the Ethics Committee of the Tianjin Medical University. The SPF grade, 3- to 4-weeks-old BALB/c nude mice were assigned to three groups (n=6 each group). The mice were implanted with A549 cell suspension in the left anterior axilla of nude mice as mentioned previously [13]. The volume of tumor cells were calculated using a formula (volume = long diameter × short diameter^2^/2). When tumor volume reached 200 mm^3^, mice were treated with vehicle control (sesame oil) and AT-101 dissolved in sesame oil (35 mg/kg/day) by oral gavage for 10 consecutive days. Until to the final time point (3 weeks), the mice were sacrificed. The xenograft tumors were then removed and fixed in formalin for preparation of paraffin-embedded sections.

### Immunohistochemistry (IHC) staining

In the IHC staining assay, the protocol of previous study was followed [14]. The following primary antibody against APE1 (Cell signaling, MA), Ki-67 (Santa Cruz, CA), E-cadherin and Vimentin (Cell signaling, MA) were used for samples staining.

### Statistical analysis

Statistical analyses were performed using SPSS (IBM SPSS Statistics 19). The Kaplan–Meier curves for PFS and OS were compared with the Breslow and Log rank test based on the survival time by median survival time (MST). Multivariate Cox proportional hazards models were performed to estimate adjusted hazard ratios (HRs) with 95% confidence intervals (95% CIs). Data was analyzed by means ± S.E. Statistical analysis was calculated using the Pearson correlation test, χ2 test, Fisher's exact test and student's t-test. Two-sided p values<0.05 were considered as differences significant.

## SUPPLEMENTARY TABLES




