# Correction: Prediction of survival prognosis of non-small cell lung cancer by APE1 through regulation of epithelial-mesenchymal transition

**DOI:** 10.18632/oncotarget.28141

**Published:** 2022-01-13

**Authors:** Xi Wei, Qing Li, Ying Li, Wei Duan, Chongbiao Huang, Xiangqian Zheng, Lei Sun, Jingtao Luo, Dong Wang, Sheng Zhang, Xiaojie Xin, Ming Gao

**Affiliations:** ^1^Department of Diagnostic and Therapeutic Ultrasonography, Tianjin Medical University Cancer Institute and Hospital, National Clinical Research Center of Cancer, Key Laboratory of Cancer Prevention and Therapy, Tianjin, China; ^2^Cancer Center, Daping Hospital and Research Institute of Surgery, Third Military Medical University, Chongqing, China; ^3^The Third Department of Breast Cancer, Tianjin Medical University Cancer Institute and Hospital, National Clinical Research Center of Cancer, Key Laboratory of Cancer Prevention and Therapy, Tianjin, China; ^4^Department of Senior Ward, Tianjin Medical University Cancer Institute and Hospital, National Clinical Research Center of Cancer, Key Laboratory of Cancer Prevention and Therapy, Tianjin, China; ^5^Department of Biochemistry and Molecular Biology, Tianjin Medical University Cancer Institute and Hospital, Tianjin, China; ^6^The Department of Otorhinolaryngology and Maxillofacial Oncology, Tianjin Medical University Cancer Institute & Hospital, Key Laboratory of Cancer Prevention and Therapy, Tianjin Cancer Institute, National Clinical Research Center of Cancer, Tianjin, China; ^7^Department of Thyroid and Cervical Tumor, Tianjin Medical University Cancer Institute and Hospital, National Clinical Research Center of Cancer, Key Laboratory of Cancer Prevention and Therapy, Tianjin, China; ^*^These authors contributed equally to this work

**This article has been corrected:** In Figure 4D, the 3rd row, 3rd panel image is an accidental duplicate of the 1st row, 2nd panel image. In addition, the figure legend for 4D has been updated to read, ‘D. A549 cells were indicated by immunofluorescence after APE1 siRNA and inhibitor AT101 treatment. (magnification: 1000×)’. The corrected Figure 4D, produced using the original data, is shown below. The authors declare that these corrections do not change the results or conclusions of this paper.

Original article: Oncotarget. 2016; 7:28523–28539. 28523-28539. https://doi.org/10.18632/oncotarget.8660

**Figure 4:**
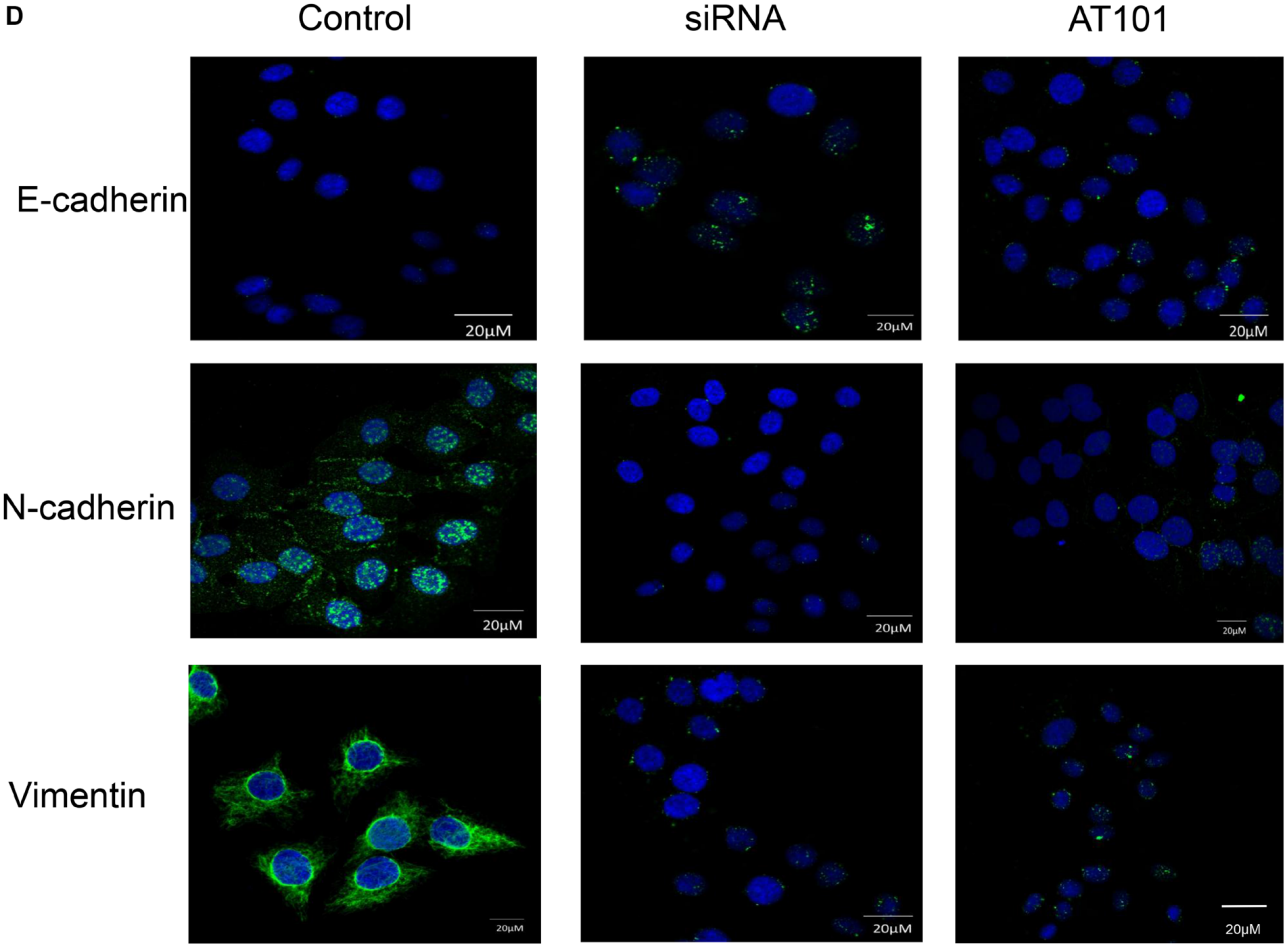
(**D**) A549 cells were indicated by immunofluorescence after APE1 siRNA and inhibitor AT101 treatment. (magnification: 1000×).

