# Supplementary material for: Prediction of survival prognosis of non-small cell lung cancer by APE1 through regulation of epithelial-mesenchymal transition
Source: Oncotarget. 2016 Apr 8;7(19):28523–39. doi: 10.18632/oncotarget.8660 (PMC5053743; doi:10.18632/oncotarget.8660)
Supplement: Supplementary file 1 [file oncotarget-07-28523-s001.pdf]

Prediction of survival prognosis of non-small cell lung cancer by APE1 through regulation of epithelial-mesenchymal transition

SUPPLEMENTARY TABLES

Supplementary Table S1: Univariate analysis of all patients with NSCLC after surgery

Characteristics	Number (%)	PFS		OS	
	423(100%)	MST(m)	*P*	MST(m)	*P*
Age(y)			0.804		0.744
<60	241	6.0		15.0	
≥60	182	6.0		16.0	
Gender			0.507		0.016*
Male	316	5.0		14.0	
Female	107	8.0		18.0	
Smoking			0.016*		0.013*
No	169	8.0		17.0	
Yes	254	5.0		15.0	
Pathological			0.210		0.018
Squamous carcinoma	120	6.0		18.0	
Adenocarcinoma	239	6.0		16.0	
Other	64	5.0		12.0	
TNM stage			0.000		0.000
II	65	12.0		23.0	
III	181	6.0		16.0	
IV	177	5.0		12.0	
Lymph node metastasis			0.000		0.000*
No	118	9.0		19.0	
Yes	305	5.0		14.0	
Distant metastasis			0.000		0.000
0	250	7.0		18.0	
1	104	6.0		16.0	
≥2	69	3.0		10.0	
Chemotherapy regimen			0.879		0.992
TP	336	6.0		16.0	
GP	87	5.0		16.0	
Toxicity			0.001		0.045
NO	253	5.0		15.0	
Grade 3–4toxicity	170	8.0		17.0	

*Breslow (Generalized Wilcoxon)
MST: median survival time; PFS: progression-free survival; OS: overall survival
m: month

Supplementary Table S2: Hazard ratios for progression-free survival (PFS) and overall survival (OS)

See supplementary File 1

Supplementary Table S3: Univariate analysis of the polymorphism of APE1-141 and expression of APE1 in NSCLC patients

		Number	PFS		OS	
APE1		123	MST(m)	*P*	MST(m)	*P*
	positive	108	6.0	0.761	14.0	0.559
	negative	15	5.0		18.0	
APE1-141		123		0.404		0.051*
	TT	62	7.0		16.0	
	GT	55	5.0		12.0	
	GG	6	8.0		14.0	

*Breslow (Generalized Wilcoxon)
MST: median survival time; PFS: progression-free survival; OS: overall survival
m: month

Supplementary Table S4: Comparison of PFS and OS for the polymorphism of APE1-141 stratified by the expression of APE1

		Number	PFS		OS	
APE1-positive		108	MST(m)	*P*	MST(m)	*P*
APE1-141	TT	54	7.0	0.512	16.0	0.037
	GT	48	5.0		12.0	
	GG	6	8.0		14.0	
APE1-negative		15		0.511		0.099
	TT	8	4.0		18.0	
	GT	7	5.0		11.0	
	GG	0	-		-	

MST: median survival time; PFS: progression-free survival; OS: overall survival
m: month

Supplementary Table S5: Comparison of PFS and OS for the expression of APE1 stratified by lymph node metastasis

		Number	PFS		OS	
Lymph node metastasis (Yes)			MST(m)	*P*	MST(m)	*P*
APE1	positive	56	4.0	0.094	12.0	0.036*
	negative	22	6.0		18.0	
Lymph node metastasis (No)						
APE1	positive	31	8.0	0.474	14.0	0.439
	negative	14	11.0		18.0	

*Breslow (Generalized Wilcoxon)
MST: median survival time; PFS: progression-free survival; OS: overall survival
m: month

Supplementary Table S6: The prediction of deacetylation on internal lysines of APE1

Peptide	Position	Score	Threshold
****MPKRGKKGA	3	17.94	0.5
*MPKRGKKGAVAE	6	3.63	0.5
MPKRGKKGAVAED	7	2.73	0.5
RTEPEAKKSKTAA	24	1.46	0.5
TEPEAKKSKTAAK	25	2.23	0.5
PEAKKSKTAAKKN	27	2.98	0.5
KSKTAAKKNDKEA	31	3.50	0.5
SKTAAKKNDKEAA	32	2.64	0.5
AAKKNDKEAAGEG	35	1.47	0.5
EDPPDQKTSPSGK	52	0.82	0.5
KTSPSGKPATLKI	58	2.58	0.5
GKPATLKICSWNV	63	1.40	0.5
RAWIKKKGLDWVK	79	0.93	0.5
LCLQETKCSENKL	98	0.63	0.5
WSAPSDKEGYSGV	125	0.86	0.5
SRQCPLKVSYGIG	141	1.29	0.5
AFRKFLKGLASRK	197	1.78	0.5
KGLASRKPLVLCG	203	2.06	0.5
IDLRNPKGNKKNA	224	1.72	0.5

MPKRGKKGAVAEDGDELRTEPEAKKSKTAAKKNDKEAAGEGPALYEDPPDQKTSPSGKPATLKICSWNVDGLR
AWIKKKGLDWVKEEAPDILCLQETKCSENKLPAELQELPGLSHQYWSAPSDKEGYSGVGLLSRQCPLKVSYGIG
DEEHDQEGRVIVAEFDSFVLVTAYVPNAGRGLVRLEYRQRWDEAFRKFLKGLASRKPLVLCGDLNVAHEEIDLRNP
KGNKKNAGFTPQERQGFGELLQAVPLADSFRHLYPNTPYAYTFWTYMMNARSKNVGWRLDYFLLSHSLLPALCD
SKIRSKALGSDHCPITLYLAL **(APE1 protein sequence)**
