# Supplementary material for: Prediction of survival prognosis of non-small cell lung cancer by APE1 through regulation of epithelial-mesenchymal transition
Source: Oncotarget. 2016 Apr 8;7(19):28523–39. doi: 10.18632/oncotarget.8660 (PMC5053743; doi:10.18632/oncotarget.8660)
Supplement: Supplementary file 2 [file oncotarget-07-28523-s002.docx]

**Table 2. Hazard ratios for progression-free survival (PFS) and overall survival (OS)**

|  | **Variables** | **Number (%)** |  |  |
| --- | --- | --- | --- | --- |
| PFS |  | 423(100) | HR (95% CI) | *P* |
|  | Smoking |  |  |  |
|  | No | 169 (11.2) | 1 (ref) |  |
|  | Yes | 254(78.5) | 1.38(1.10-1.73) | 0.005 |
|  | TNM stage |  |  |  |
|  | II | 65(3.0) | 1 (ref) |  |
|  | III | 181(43.5) | 1.60 (1.16-2.20) | 0.004 |
|  | IV | 177(53.5) | 2.02(1.46-2.80) | 0.000 |
|  | Lymph node metastasis |  |  |  |
|  | No | 118(40.3) | 1 (ref) |  |
|  | Yes | 305(59.7) | 1.67(1.30-2.14) | 0.000 |
|  | Distant metastasis |  |  |  |
|  | 0 | 250(40.1) | 1 (ref) |  |
|  | 1 | 104(59.9) | 1.01(0.67-1.54) | 0.946 |
|  | ≥2 | 69 | 1.68(1.07-2.67) | 0.025 |
|  | Toxicity |  |  |  |
|  | NO | 253 | 1 (ref) |  |
|  | Grade 3–4toxicity | 170 | 0.69(0.55-0.87) | 0.001 |
| OS | Gender |  |  |  |
|  | Female | 240(50.6) | 1 (ref) |  |
|  | Male | 234(49.4) | 1.16(0.91-1.49) | 0.232 |
|  | Smoking |  |  |  |
|  | No | 169 (11.2) | 1 (ref) |  |
|  | Yes | 254(78.5) | 1.10(0.89-1.38) | 0.383 |
|  | Pathological |  |  |  |
|  | Other | 64 | 1 (ref) |  |
|  | Adenocarcinoma | 239(12.5) | 0.77(0.56-1.04) | 0.091 |
|  | Squamous carcinoma | 120(85.0) | 0.62 (0.44-0.87) | 0.006 |
|  | TNM stage |  |  |  |
|  | Ⅱ | 65 | 1 (ref) |  |
|  | Ⅲ | 181 (3.0) | 1.55(1.11-2.16) | 0.010 |
|  | Ⅳ | 177 (43.5) | 2.34(1.68-3.26) | 0.000 |
|  | Lymph node metastasis |  |  |  |
|  | No | 118 | 1 (ref) |  |
|  | Yes | 305 | 1.37(1.07-1.74) | 0.012 |
|  | Distant metastasis |  |  |  |
|  | 0 | 250 | 1 (ref) |  |
|  | 1 | 104 | 1.36(1.05-1.76) | 0.020 |
|  | ≥2 | 69 | 2.32(1.73-3.13) | 0.000 |
|  | Toxicity |  |  |  |
|  | NO | 170(19.6) | 1 (ref) |  |
|  | Grade 3–4toxicity | 253(80.4) | 0.78(0.62-0.97) | 0.026 |

***P*<0.001

MST: median survival time; PFS: progression-free survival; OS: overall survival

m: month
